# Hypoxic/ischemic hits predispose to necrotizing enterocolitis in (near) term infants with congenital heart disease: a case control study

**DOI:** 10.1186/s12887-020-02446-6

**Published:** 2020-12-07

**Authors:** Martin van der Heide, Mirthe J. Mebius, Arend F. Bos, Marcus T.R. Roofthooft, Rolf M.F. Berger, Jan B.F. Hulscher, Elisabeth M.W. Kooi

**Affiliations:** 1 Division of Neonatology, University of Groningen, University Medical Center Groningen, Beatrix Children’s Hospital, Hanzeplein 1, 9713 GZ Groningen, The Netherlands; 2 Division of Pediatric Cardiology, University of Groningen, University Medical Center Groningen, Beatrix Children’s Hospital, Groningen, The Netherlands; 3 Department of Surgery, Division of Pediatric Surgery, University of Groningen, University Medical Center Groningen, Groningen, The Netherlands

**Keywords:** Necrotizing enterocolitis, congenital heart disease, (near) term infants, diastolic blood pressure, Apgar score, hypoxic/ischemic hits

## Abstract

**Background:**

Necrotizing enterocolitis (NEC) is a devastating disease that is relatively frequently diagnosed in term infants with congenital heart disease (CHD), compared with term infants without CHD, in whom NEC is rare. The exact pathogenesis of NEC in term infants with CHD is unknown, but it is hypothesized that ischemia of the intestines plays a pivotal role. We aimed to explore whether (near) term CHD infants, who develop NEC, exhibit more clinical signs of hypoxia/ischemia and low body perfusion directly after birth and during the first 48 hours after admission to the neonatal intensive care unit, when compared with (near) term CHD infants who did not develop NEC.

**Methods:**

956 infants with CHD born after ≥ 35 weeks of gestational age were retrospectively reviewed for this case-control study between January 1999 and February 2020. We included infants with radiographically confirmed pneumatosis intestinalis and controls matched by type of CHD. Seven infants were diagnosed with transposition of the great arteries, six with left and four with right ventricular outflow tract obstruction. Several parameters suggestive of (relative) hypoxia/ischemia were used for analyses.

**Results:**

We included sixteen CHD infants with NEC and selected sixteen controls. There were no significant demographic differences between both groups. Apgar score at one and five minutes (median [IQR]) were lower in infants who developed NEC compared with control infants (8 [7-8]) vs. (9 [8-9], *P* = .011) and (8 [8-9]) vs. (9 [9-10], *P* = .009). A higher proportion of infants with NEC required respiratory support in the delivery room (11(69) vs. 2(13), *P* = .001). The (median [IQR]) diastolic blood pressure on the second day after admission (39 mmHg [34–42], vs. 43 mmHg [37–51], *P* = .112) and lowest (median [IQR]) pH in the 48 hours after admission (7.24 [7.17–7.35] vs. 7.38 ([7.27–7.43], *P* = .157) were not significantly lower in NEC infants but both demonstrated a similar direction towards (relative) hypoxia/ischemia in NEC infants.

**Conclusions:**

Our clinical results support a hypoxic/ischemic pathophysiology of NEC in (near) term CHD infants, with lower Apgar scores, more respiratory support in the delivery room and a tendency towards a lower diastolic blood pressure and pH in CHD infants who develop NEC.

## Background

Necrotizing enterocolitis (NEC) is a devastating disease that mainly affects preterm infants. Although rare, NEC also occurs in (near) term infants, where congenital heart disease (CHD) is known to be a risk factor [1–3]. Of all term infants with NEC, up to one third has been reported to have a CHD while 3.3% of the infants with a CHD have been reported to develop NEC [1, 4]. Depending on the type of CHD, the incidence of NEC may even approach the incidence in very low birth weight infants [5].

Although NEC is diagnosed more frequently in term infants with CHD, it is still unclear why these term infants with CHD have an increased risk of developing NEC compared to term infants without CHD and if and in which way pathogenesis may be different from preterm NEC. Previous studies report differences in age of onset, [2] NEC localisation in the intestines, [6] and severity of NEC between infants with and without CHD [7]. Our group previously demonstrated that, in preterm infants without CHD, NEC predominantly occurred in the small intestines, whereas in CHD infants the predominant NEC localisation was in the colon. The colon is probably more susceptible for ischemia as it relies on the most distal branches of its vascular supply. These watershed zones are at highest risk of ischemia in times of reduced blood supply [6]. These observations support the hypothesis that intestinal ischemia plays a primary role in the development of NEC in infants with CHD. However, the exact mechanism that leads to this ischemic NEC in CHD infants remains unclear.

As most CHD infants already develop NEC in the first week of life, these infants may be more exposed to hypoxia and ischemia of the intestines in the first days after birth [2, 8]. During these first days, CHD infants are at increased risk for ischemia during both delivery and the transition from fetal to neonatal circulation. Also, ischemia of the intestines due to a ductal steal phenomenon, has been suggest in term CHD infants, characterized by a retrograde diastolic blood flow in the abdominal aorta due to an unrestricted left-to-right shunt through an open ductus arteriosus, accompanied by lower diastolic blood pressures [1, 9]. In infants with duct-dependent pulmonary or systemic circulation, treated with prostaglandins, retrograde diastolic backflow is often present [10].

We hypothesize that (near) term CHD infants who develop NEC have experienced multiple hits of relative hypoxia/ischemia in the first days after birth. Therefore, we aimed to explore whether (near) term CHD infants who later on develop NEC exhibit more clinical signs of hypoxia/ischemia and low body perfusion directly after birth and during the first 48 hours after admission to the neonatal intensive care unit (NICU), when compared with (near) term CHD infants who did not develop NEC.

## Methods

### Patient population

In this retrospective case-control study, we included all infants with a CHD and a gestational age of 35 weeks or more who were admitted to the tertiary congenital heart center of the University Medical Center Groningen between January 1999 and February 2020. We excluded infants who developed NEC before or at the day of admission to the NICU. In infants with clinical signs of NEC, we confirmed NEC diagnosis by pneumatosis intestinalis on abdominal radiograph, classified by the clinician and radiologist, and confirmed by two authors (MH, EK) [11, 12]. As controls, we selected the subsequently born, or if unavailable the previous born infant with a similar type of CHD who did not develop NEC with available registered values of SpO_2_, heart rate and blood pressure on both days after admission to the NICU.

### Clinical variables

Various baseline parameters were collected including sex, inborn, caesarian section, birth weight, small for gestational age status and percentiles of birth weight according to Dutch reference values, [13] head circumference, gestational age, cyanotic or non-cyanotic CHD, antenatal diagnosis of the CHD, percentage of mother’s milk, and amount of feeding (ml/kg). We used noninvasively collected diastolic blood pressure values for analyses during the first 48 hours after admission. Invasively collected diastolic blood pressure values, measured at the right radial artery, were used when noninvasive diastolic blood pressure values were not available. Mean diastolic blood pressure was calculated from patients’ records for the first and second 24 hours after admission. Of note, from infants who already developed NEC on the second day after admission, only the data collected during the first day after admission were analyzed. Furthermore, we assessed the following parameters associated with tissue hypoxia/ischemia and/or low body perfusion: Apgar score at 1 and 5 minutes, need for respiratory support in the delivery room, post-ductal SpO_2_ (Nellcor®, Medtronic, Dublin, Ireland), daily mean heart rate, (either documented manually in the daily charts or when available derived from an offline stored digital database), first hemoglobin value after admission, lowest arterial or capillary pH value in the 48 hours after admission, metabolic acidosis (defined as a pH < 7.3 and bicarbonate < 22), respiratory support, systolic blood pressure and treatment with prostaglandin E_1_ and inotropes.

Echocardiographic evaluation was performed in all infants during the first day of admission by the attending pediatric cardiologist or a trained pediatric ultrasonographist, and offline confirmed by one author (MR). Echocardiographic parameters collected included internal ductal diameter, ductal flow patterns, LA:Ao-ratio or LA:pa-ratio for infants with a transposition of the great arteries, left ventricle end diastolic diameter, diastolic backflow in the descending aorta, and diastolic forward blood flow in the pulmonary arteries.

Information about surgical procedures in the first 48 hours after admission or before NEC development was collected. We obtained all parameters from the digital hospital information system and patients’ records. This study was approved by the ethical review board of the University Medical Center Groningen.

### Statistical Analysis

Standard statistical tests to detect differences between NEC infants and infants without NEC were used for parametric and nonparametric data, including independent t-test and Mann Whitney U test, expressed as mean ± SD and median (IQR) respectively. For nominal and categorical variables, we used Chi square tests and Fisher’s exact tests expressed as number and percentage. Two-tailed *P*-values of less than 0.05 were considered statistically significant. Because we matched every NEC infant with a control infant, we excluded the data of the matched infant in the case of missing values. For each parameter that we compared, if not all data were available for all patients, we chose to only include patients with available data in both groups. Statistical Package for Social Sciences (IBM SPSS Statistics 22, IBM Corp., Armonk, NY, USA) was used for statistical analyses.

## Results

### Patient characteristics

**Fig. 1:**
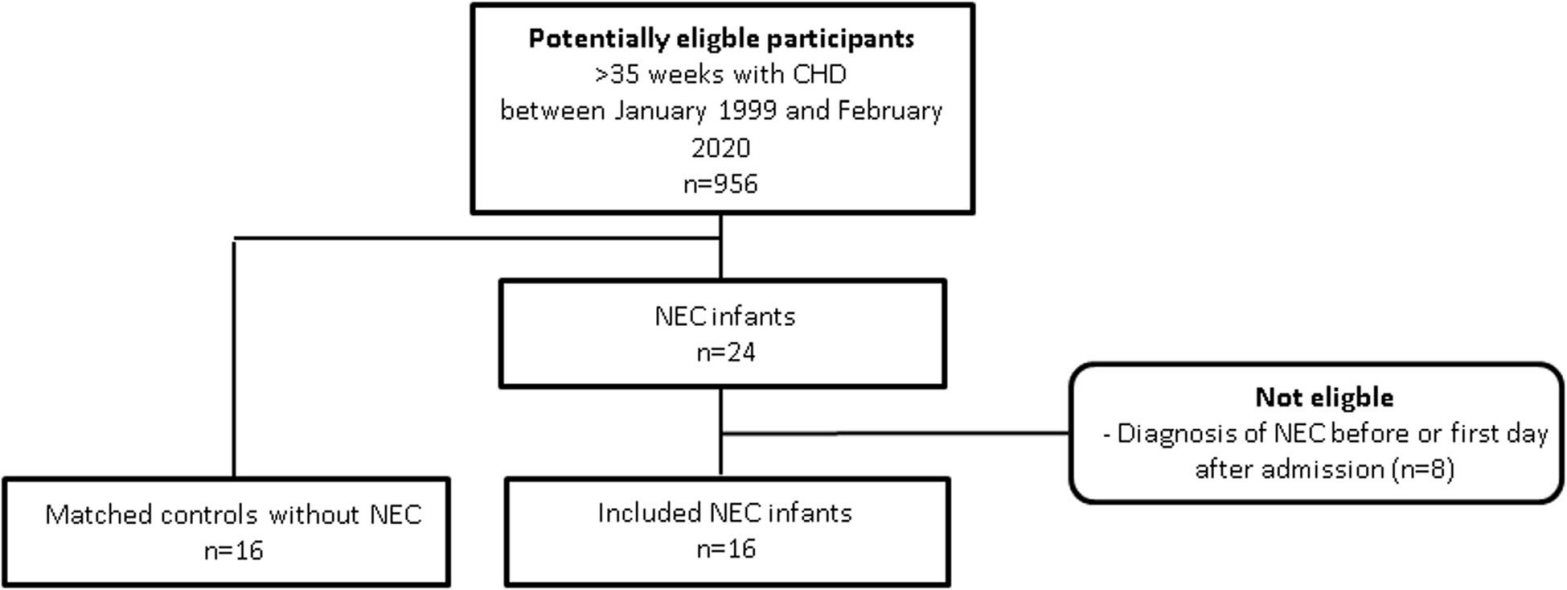
Flow diagram of the study. CHD: congenital heart disease. NEC: necrotizing enterocolitis

Out of 956 eligible CHD infants admitted to our NICU between January 1999 and February 2020, 24 infants (2.5%) developed NEC. Eight of these infants were excluded as they were diagnosed with NEC before or on the first day of admission. Therefore, sixteen infants with NEC and sixteen controls matched for type of CHD were included in the analysis (Fig. 1). For the second day after admission, ten infants with NEC and ten controls remained for analyses because three infants already developed NEC on the second day, and we were unable to retrieve SpO_2_, heart rate and blood pressure values on the second day after admission of three NEC infants.

Type of CHD and Bell stage of the infants are reported in Table 1. Of the included sixteen infants who developed NEC, ten were diagnosed with a cyanotic heart lesion. Six infants developed NEC Bell stage 2A, four Bell stage 2B and six Bell stage 3B. NEC was confirmed at a median postnatal age of 5.5 days (3–11 days) which was 4.5 (2–11) days after NICU admission.

**Table 1:** Type of CHD and Bell stage for infants with NEC and their matched controls

	NEC	Control
Bell stage	Type of CHD	Type of CHD
3B	Tetralogy of Fallot	Tetralogy of Fallot
3B	IAA, DORV and AVSD	IAA, PFO, VSD and small aortic valve
3B	Pulmonary valve stenosis	Pulmonary valve stenosis
2A	Tetralogy of Fallot with pulmonary atresia	Tetralogy of Fallot with pulmonary atresia
2A	TGA with DORV, VSD and PFO	TGA with DORV, VSD and tricuspid insufficiency
3B	TGA with VSD	TGA with VSD
3B	TGA with AVSD and small LV	TGA with VSD and ASD
2A	IAA with VSD	IAA with VSD
2A	IAA with VSD	IAA with VSD and hypoplastic aortic valve
2B	CoA with TGA, hypoplastic aortic arch, DORV, VSD and ASD	CoA with hypoplastic aortic arch, DORV, VSD and ASD
3B	TGA	TGA
2B	Tetralogy of Fallot	Tetralogy of Fallot
2B	TGA with DORV and VSD	TGA with DORV and VSD
2A	CoA with VSD	CoA with VSD
2A	CoA with hypoplastic aortic arch, VSD and ASD	CoA with hypoplastic aortic arch and VSD
2B	TGA with VSD	TGA with VSD

*IAA* interrupted aortic arch, *DORV* double outlet right ventricle, *AVSD* atrioventricular septal defect, *PFO* patent foramen ovale, *VSD* ventricular septal defect, *TGA* transposition of the great arteries, *LV* left ventricle, *ASD* atrial septal defect, *CoA* Coarctation of the aorta

There were no significant differences between both groups regarding sex, gestational age, head circumference, birth weight, SGA status, percentiles of birth weight, inborn, caesarian section, and age of admission to the NICU. All infants received either formula or mother’s milk in the first 24 hours after admission. The percentage of mother’s milk was not different between NEC infants and control infants in the first 24 hours after admission (0% [0–0%], *n* = 10, vs. 7% [0–39%], *n* = 10, *P* = .183). Moreover, the amount of feedings was not different between NEC infants and control infants during the first 24 hours after admission (15ml/kg [8–20], *n* = 10, vs. 23ml/kg [11–37], *n* = 10, *P* = .326). Baseline characteristics are reported in Table 2.

**Table 2:** Baseline characteristics

	NEC (*n* = 16)	Control (*n* = 16)	*P*
Sex (male) (%)	9 (56)	9 (56)	1.00
Gestational age (weeks)	38.6 ± 1.9	39.1 ± 1.7	0.37
Head circumference (cm)^a^	33.5 (33.0–36.0)	34.6 (32.0–37.0)	0.25
Birth weight (grams)	2937 ± 701	3323 ± 546	0.14
Small for gestational age status (%)	5 (31)	1 (6)	0.07
Percentiles of birth weight	27 (1–58)	41 (20–77)	0.22
Inborn (%)	10 (63)	9 (56)	0.72
Caesarian section (%)	6 (38)	2 (13)	0.22
Mother’s milk (%)^b^	0 (0–0)	7 (0–39)	0.18
Amount of feeding (ml/kg)^b^	15 (8–20)	23 (11–37)	0.33
Antenatal diagnosis of CHD (%)	8 (50)	9 (56)	0.72
Age at admission to NICU (days)	0 (0–1)	0 (0–1)	0.77

Data are shown as either *n* (%) for categorical variables and mean ± standard deviation or median (IQR) for continuous variables. ^a^: in both groups 14 infants were used for analysis. ^b^: in both groups 10 infants were used for analysis. *CHD* congenital heart disease

Rashkind balloon atrial septostomy procedure was performed in three infants before the development of NEC and in two control infant on the first day after admission to the NICU. Furthermore, balloon dilatation of the pulmonary valve was performed in one control infant the second day after admission to the NICU. In the other infants, no invasive interventions were performed between day of admission and the development of NEC.

### Parameters associated with hypoxia/ischemia or low body perfusion

In Table 3 we present all parameters associated with (relative) hypoxia/ischemia or low body perfusion. Apgar scores at 1 minute were statistically lower in the group with NEC (8 [7-8]) than in the control group (9 [8-9], *P* = .011). Moreover, Apgar scores at 5 minutes were also significantly lower in the group with NEC (8 [8-9]), than in the control group (9 [9-10], *P* = .009). A higher proportion of infants who developed NEC required respiratory support in the delivery room (11 (69) vs. 2 (13), *P* = .001). Of these eleven infants with NEC, two infants required invasive ventilation, seven infants required continuous positive airway pressure (CPAP), and two infants required oxygen support. In the control group none of the infants required invasive ventilation, one infant required CPAP, and one infant required oxygen support. The lowest pH in the first 48 hours after admission to the NICU was not different between infants with and without NEC (7.24 [7.17–7.35], *n* = 9 vs. 7.39 [7.27–7.43], *n* = 9, *P* = .157). First Hb-value after admission did not differ between the NEC group (9.2 mmol/l [8.5–10]) and controls (10.8 mmol/l [8.4–11.6], *n* = 9, *P* = .22). SpO_2_ and heart rate were not different between NEC infants and controls on both the first and second day after admission (Table 3). As we started measuring lactate levels on a regular basis after the inclusion period of this study, we did not have sufficient lactate values available for the current study population. All echocardiographic parameters and other parameters associated with hypoxia/ischemia or low body perfusion were similar between NEC and control infants (Table 3).

**Table 3:** Variables associated by low body perfusion and hypoxia for infants before NEC development

	NEC	n	Control	n	*P*
Apgar score at 1 minute ^a^	8 (7–8)	12	9 (8–9)	12	0.01
Apgar score at 5 minutes ^a^	8 (8–9)	12	9 (9–10)	12	0.009
Respiratory support in the delivery room (%) ^a^	11 (69)	16	2 (13)	16	0.001
Diastolic blood pressure 0–24 h (mm Hg) ^a^	42 (34–44)	16	40 (38–48)	16	0.49
Diastolic blood pressure 24–48 h (mm Hg)^b^	39 (34–42)	10	43 (37–51)	10	0.11
Systolic blood pressure 0–24 h (mm Hg) ^a^	68 (63–76)	16	72 (66–77)	16	0.37
Systolic blood pressure 24–48 h (mm Hg)^b^	67 (62–74)	10	70 (68–80)	10	0.29
First Hb measurement (mmol/L)^b^	9.3 (8.7–10.1)	11	10.8 (9.1–11.6)	11	0.26
Lowest pH measurement ^a^	7.24 (7.17–7.35)	9	7.38 (7.27–7.43)	9	0.16
Metabolic acidosis	4 (44)	9	2 (22)	9	0.62
Post-ductal SpO_2_ 0–24 h ^a^	91 (89–96)	13	93 (89–94)	13	0.58
Post-ductal SpO_2_ 24–48 h ^b^	93 (89–95)	12	93 (90–95)	12	0.88
Heart rate 0–24 h ^a^	143 (142–157)	11	146 (134–154)	11	0.49
Heart rate 24–48 h^b^	149 (138–155)	8	140 (133–150)	8	0.40
Respiratory support 0–24 h (%) ^a^	9 (56)	16	10 (63)	16	0.70
Respiratory support 24–48 h (%)^b^	4 (50)	8	4 (50)	8	1.00
Prostaglandin E_1_ 0–24 h (%) ^a^	8 (50)	16	11 (69)	16	0.28
Prostaglandin E_1_ 24–48 h (%)^b^	4 (44)	9	5 (56)	9	1.00
Inotropes (%) ^a^	2 (13)	16	1 (6)	16	1.00
Internal ductal diameter (mm) ^a^	2.8 (1.9–3.4)	11	3.3 (3.0-3.6)	11	0.17
LA:Ao-ratio ^a^	1.7 (1.5–2.3)	9	1.8 (1.4–1.9)	9	0.51
LVEDD (mm) ^a^	16.0 (11.2–20.0)	10	15.5 (14.2–18.1)	10	0.94
Diastolic backflow in the descending aorta (%) ^a^	6 (55)	11	5 (46)	11	0.67
Diastolic forward blood flow in the pulmonary arteries (%) ^a^	7 (70)	10	7 (70)	10	1.00

Data are shown as either *n* (%) for categorical variables and mean ± standard deviation or median (IQR) for continuous variables. ^a^: Maximum of 16 infants used for the variable. ^b^: Maximum of 13 infants used for the variable due to the development of NEC in 3 infants. LA:Ao-ratio: left atrium-aortic root ratio. LVEDD: Left ventricular end diastolic diameter

Median diastolic blood pressure did not differ significantly between the NEC group (42 (34–44) mm Hg, *n* = 16) and the control group (40 (38–48) mm Hg, *n* = 16) on both the first day after admission to the NICU (*P* = .49) and the second day after admission (39 (34–42) mm Hg, *n* = 10) vs. (43 (37–51) mm Hg, *n* = 10, *P* = .11). The increase in diastolic blood pressure between day one and two was not different between NEC infants and control infants (1 [-4-5] mm Hg vs. 6 [1–6] mm Hg, *P* = .112). Systolic blood pressure was not different on both days between both groups (Table 3).

## Discussion

In this study we demonstrated that (near) term CHD infants who develop NEC had lower Apgar scores at one and five minutes and required more respiratory support in the delivery room than infants without NEC. Although not reaching statistical significance, the lowest pH in the first 48 hours after admission and the diastolic blood pressure on the second day after admission tended to be lower in the NEC infants when compared to the matched controls, which might be clinically relevant.

The lower Apgar scores, higher incidence of respiratory support in the delivery room and the tendency towards a clinically relevant lower pH and diastolic blood pressure, which indirectly may reflect relative hypoxia/ischemia, suggest a hypoxic/ischemic etiology of NEC in (near) term CHD patients. Although our results were found in a relative small sample, these results may give insights in the development of NEC in (near) term CHD infants. Reduction of these hypoxic/ischemic events may potentially reduce the risk of NEC, but this has to be confirmed in a prospective study.

To the best of our knowledge we are the first to suggest that several relative hypoxic/ischemic circumstances may already occur before and during the first 48 hours after admission to the NICU in (near) term CHD infants who subsequently develop NEC. We demonstrated that CHD infants who developed NEC had lower Apgar scores at one and five minutes compared with matched control infants. Others reported no association between lower Apgar score and NEC in (near) term CHD infants [1, 10]. This could be caused by the fact that we only included infants who developed NEC before surgery while others also included infants who developed NEC after surgery, limiting relative smaller perinatal effects. In contrast with the study by Carlo et al. who reported that a diastolic steal phenomenon, defined as retrograde flow in the abdominal aorta during diastole, may predispose to NEC in CHD patients, we found no signs of ductal steal [10]. In our study we did not find a higher incidence of retrograde diastolic flow in the abdominal aortae nor a statistically significant lower diastolic blood pressure in NEC infants which may have indicated ductal steal. This may have been caused by a lack of power. Although not reaching statistical significance, the difference in diastolic blood pressure and pH in our study could be clinically relevant as both demonstrated the same direction towards (relative) hypoxia/ischemia in NEC infants. Therefore, we need to repeat this study in a prospective multicenter study.

Our findings support the hypothesis that NEC in term CHD infants has a hypoxic/ischemic etiology. The first potential hypoxic/ischemic event which CHD infants may experience is during delivery. As both Apgar score at one and five minutes were lower in infants who developed NEC and a higher proportion of infants who develop NEC required respiratory support directly after birth, their intestines may have been exposed to relatively more hypoxia/ischemia already in the very first minutes of life. However, as the Apgar score at one and five minutes were eight in the NEC group, this first potential hypoxic/ischemic event hardly seems a major contributor to NEC development [13, 14]. Moreover, it is unknown if this one-point lower Apgar score was caused by central cyanosis or other components of the Apgar score because these data could not be retrieved from the charts. The need of respiratory support in the delivery room suggests that hypoxia may have been present in NEC infants and may also have induced a relative hypoxic/ischemic episode which, in combination with an already higher degree of hypoxia due to a lower Apgar score, may affect the intestines and induce NEC development. The high incidence of respiratory support in the NEC group might also be explained by aerophagia. We speculate that aerophagia, which is caused by CPAP, may increase pressure to the intestinal wall which subsequently may hamper intestinal wall perfusion. In combination with an already suboptimal perfusion due to the CHD, this may further induce ischemia. Some studies in preterm infants indeed showed that prolonged CPAP increased the risk of NEC and that infants with CPAP failure have a higher risk of NEC compared to infants with invasive ventilation [14, 15]. However, this all remains highly speculative.

We could not confirm previous findings of a lower diastolic blood pressure in infants developing NEC. Similarly, we did not find a difference in the incidence of retrograde diastolic flow between infants who developed NEC (55%) and control infants (46%) [10]. However, in our study diastolic blood pressure and pH showed the same direction towards hypoxia/ischemia in NEC infants. A previous study reported that infants with hypoplastic left heart syndrome were at highest risk of developing NEC.[1] Surprisingly, in our cohort of 956 infants, none of the infants with hypoplastic left heart syndrome developed NEC, but seven were diagnosed with transposition of the great arteries and six with aortic arch obstruction. This different study population may also explain different findings with previous studies next to the fact that we only included infants who developed NEC before they had cardiac surgery, while others included infants who developed NEC both before and after cardiac surgery [1, 10].

Due to low incidence of NEC in CHD infants and therefore a low number of inclusions, we were not able to demonstrate a statistically significant difference in pH, SpO_2_ and time to first prostaglandin administration. However, the non-significant difference in the lowest pH of 0.15 could be clinically relevant and could reach significance in a larger group. SpO_2_ was also not different in the first days after admission between NEC infants and control infants. However, as the SpO_2_ value used for analysis was the mean of all SpO_2_ values measured over 24 hours, undetected episodes of hypoxia may have occurred. Moreover, SpO_2_ measurements at an extremity may fail to detect hypoxia in the intestines due to low abdominal perfusion.

In summary, lower Apgar scores, a higher proportion of respiratory support, and a tendency towards potentially clinically relevant lower pH and diastolic blood pressure suggest a pathogenesis of repetitive relative tissue hypoxia/ischemia in (near) term CHD infants in the first days after birth leading up to NEC. This seems in part different from the pathophysiology of preterm NEC, mainly caused by other factors such as immaturity of the intestines, and a different microbiome [16]. Moreover, in preterm NEC, tissue hypoxia/ischemia is less extended and differently located than in NEC of CHD infants [6].

We recognize several limitations of our study. In this case-control study we matched infants based on type of CHD to limit selection bias due to the retrospective nature of this study. Although it proved to be difficult to match infants who developed NEC with infants of the control group with similar CHD, due to many rare combinations of types of CHD, functional echocardiographic parameters did not differ between NEC infants and control infants. Therefore it seems that both groups were properly matched. However, we did not match infants based on cardiac interventions such as balloon atrioseptectomy which could have affected our results. Second, in our study, SGA status, birth weight and birth weight percentiles all showed similar directions that NEC infants were smaller than control infants. Although some studies showed an association between birth weight and diastolic blood pressure in term infants [17, 18] this association is probably confounded by the sicker infants [19, 20]. The question remains, however, if this potential lower birth weight may have contributed to the development of NEC and if so, if this is due to insufficient placenta function in utero, known to predispose to ischemic intestines [21]. Third, we were unable to identify in retrospect at which extremity blood pressure values were measured. This could have affected our results. Fourth, as we tested various variables we may need to correct for multiple testing. However, we did not correct for multiple testing in order to find all potential associations with signs of relative hypoxia/ischemia. Finally, we were unable to validly retrieve data from referring centers of the infants that already had NEC on arrival or soon after. Therefore we had to exclude these infants from the analyses. To overcome this limitation, we advise that new studies into (near) term CHD infants with NEC should be conducted in a multicenter study, i.e. through an official NEC network, using digital patient records when possible. Although we included infants in a period of 20 years in a tertiary referral center, our relatively small sample size could have induced type II errors and limited the statistical power of this study.

The next step would be to prospectively confirm in a multicenter study whether low body perfusion and hypoxia/ischemia are indeed major contributing factors in the development of NEC by measuring other parameters associated to intestinal hypoxia/ischemia, such as regional splanchnic oxygen saturation measured by Near-infrared spectroscopy [22, 23].

## Conclusions

Our results suggest a perinatal hypoxic/ischemic pathophysiology of NEC in (near) term CHD infants with potential relative hypoxic/ischemic events. We now need to confirm our findings and prospectively investigate whether maintaining adequate abdominal perfusion and oxygenation in term CHD infants, prevents NEC development.

## Data Availability

The datasets used and/or analyzed during the current study are available from the corresponding author on reasonable request.

## Abbreviations

CHD: Congenital heart disease
NEC: Necrotizing enterocolitis
NICU: Neonatal intensive care unit
IAA: Interrupted aortic arch
DORV: Double outlet right ventricle
AVSD: Atrioventricular septal defect
PFO: Patent foramen ovale
VSD: Ventricular septal defect
TGA: Transposition of the great arteries
LV: Left ventricle
ASD: Atrial septal defect
CoA: Coarctation of the aortae
SD: Standard deviation
IQR: Interquartile range
